# A Liquid Chromatography – Tandem Mass Spectrometry Approach for the Identification of Mebendazole Residue in Pork, Chicken, and Horse

**DOI:** 10.1371/journal.pone.0169597

**Published:** 2017-01-13

**Authors:** Ji Sun Lee, Soo Hee Cho, Chae Mi Lim, Moon Ik Chang, Hyun Jin Joo, Hojae Bae, Hyun Jin Park

**Affiliations:** 1 Imported Food Analysis Division, Seoul Regional Food and Drug Administration, 212 Mokdongjungang-ro, Yangcheon-Gu, Seoul, Republic of Korea; 2 Department of Biotechnology, College of Life Sciences and Biotechnology, Korea University, 5-Ka, Anam-Dong, Sungbuk-Gu, Seoul, Republic of Korea; 3 Korea Health Supplements Association Sub. Korea Health Supplements Institute, B-dong 101, 700 Daewangpangyo-ro, Bundang-Gu, Seongnam-si, Gyeonggi-do, Republic of Korea; 4 Ministry of Food and Drug Safety, 187 Osongsaengmyeong2(i)-ro, Osong-eup, Heungdeok-gu, Chungju-si, Chungcheonbuk-do, Republic of Korea; 5 Pesticide and Veterinary Drug Residues Division, Food Safety Evaluation Department, National Institute of Food and Drug Safety Evaluation, Ministry of Food and Drug Safety, 187 Osongsaengmyeong2(i)-ro, Osong-eup, Heungdeok-gu, Chungju-si, Chungcheonbuk-do, Republic of Korea; 6 College of Animal Bioscience and Technology, Department of Bioindustrial Technologies, Konkuk University, Hwayang-dong, Kwangjin-gu, Seoul, Korea; 7 Department of Packaging Science, Clemson University, Clemson, SC, United States of America; Indian Institute of Science, INDIA

## Abstract

A confirmatory and quantitative method of liquid chromatography-tandem mass spectrometry (LC-MS/MS) for the determination of mebendazole and its hydrolyzed and reduced metabolites in pork, chicken, and horse muscles was developed and validated in this study. Anthelmintic compounds were extracted with ethyl acetate after sample mixture was made alkaline followed by liquid chromatographic separation using a reversed phase C_18_ column. Gradient elution was performed with a mobile phase consisting of water containing 10 mM ammonium formate and methanol. This confirmatory method was validated according to EU requirements. Evaluated validation parameters included specificity, accuracy, precision (repeatability and within-laboratory reproducibility), analytical limits (decision limit and detection limit), and applicability. Most parameters were proved to be conforming to the EU requirements. The decision limit (CCα) and detection capability (CCβ) for all analytes ranged from 15.84 to 17.96 μgkg^-1^. The limit of detection (LOD) and the limit of quantification (LOQ) for all analytes were 0.07 μgkg^-1^ and 0.2 μgkg^-1^, respectively. The developed method was successfully applied to monitoring samples collected from the markets in major cities and proven great potential to be used as a regulatory tool to determine mebendazole residues in animal based foods.

## Introduction

Mebendazole (MEB), a benzimidazole anthelmintic, is widely used in veterinary medicine [1, 2]. This compound is an orally active broad-spectrum anthelmintic that is effective against numerous species of nematodes and cestodes in the gastrointestinal track of animals [3]. Many benzimidazole drugs have been proven to be safe due to their greater selective affinity for parasitic tubulin than for mammalian tissues. Nonetheless, some toxic effects such as antimitotic activity (teratogenicity, genotoxicity) do occur in target species [4]. Therefore, high levels of residues in animal tissues and products are of great concern. To protect the health of consumers, EU has established the maximum residue limits (MRLs) for MEB residues in animal tissues. Marker residue of MEB is defined as the sum of MEB, its reduced metabolite (RMEB) hydroxymebendazole, and its hydrolyzed metabolite (HMEB) aminomebendazole by EU regulation [5] (Table 1). In 2012, the Ministry of Food and Drug Safety in Korea also established MRLs for MEB and its metabolites in muscle, fat, liver, and kidney of pork, chicken, and horse at 60, 60, 400, and 60 μgkg^-1^, respectively. However, the standard analytical method for MEB residues using dimethylsulphoxide (DMSO) extraction followed by purification with solid phase extraction (SPE) depicted in Korean Food Standards Codex resulted in low HMEB recovery rate from edible tissues of pork [6]. Therefore, to effectively control MEB residues in animal products, a confirmatory and quantitative method capable of detecting the complete range of marker residues is urgently needed.

**Table 1:** Maximum residue limits (MRLs) of mebendazole residues.

Pharmacologically Active Substance	Marker Residue	Animal Species	MRLs	Target Tissues	Therapeutic Classification
Mebendazole^*^	Sum of mebendazole, methyl(5-(1-hydroxy, 1-phenyl)methyl-1H-benzimidazol-2-yl) carbamate and (2-amino-1H-benzimidazol-5-yl) phenylmethanone, expressed as mebendazole equivalents	• Ovine • Caprine • Equidae	• 60 μg/kg • 60 μg/kg • 400 μg/kg • 60 μg/kg	• Muscle • Fat • Liver • Kidney	Antiparasitic agents /Agents against endoparasites

^*^ Not for use in animals from which milk is produced for human consumption.

Various determination methods for MEB in animal tissues have been developed and described in the literature. Most of them use HPLC with ultraviolet (UV) [3, 7, 8] or fluorescence (FL) [9] detection. However, these detectors have low sensitivity and specificity. To enhance the detection level, the combination of liquid chromatography and mass spectrometric detection for the determination of MEB residues has become a popular analytical technique for sensitive and selective detection of MEB residue in complex biological matrices [1, 10, 11]. In addition, liquid-liquid extraction (LLE) with additional clean-up and SPE are often used for sample preparation in food-producing animals. For instance, Huan *et al* and Dongmei *et al* have described a HPLC method with SPE to analyze benzimidazoles residues in bovine milk, grass carp, and shrimp [3, 12]. In other studies, Austin *et al* [13] and Shu-Chu *et al* [8] have reported a method for the determination of benzimidazoles in livestock using HPLC with matrix solid phase dispersion (MSPD) and LLE extraction [13]. However, methods capable for simultaneous determination of MEB, RMEB, and HMEB in chicken, horse, and pork muscles by LC-MS/MS and LLE have not been developed.

In this study, we report the development and validation of a quantitative confirmatory assay with LC-MS/MS for the determination of MEB and its metabolites in pork, chicken, and horse muscles. The newly developed LC-MS/MS method was applied to monitor the MEB residues in muscles of pork, chicken, and horse of which the MRLs for MEB and its metabolites have been establish in 2012 by Korean Ministry of Food and Drug Safety. The confirmatory method was validated according to 2002/657/EC [14].

## Materials and Methods

### Materials

**Fig 1:**
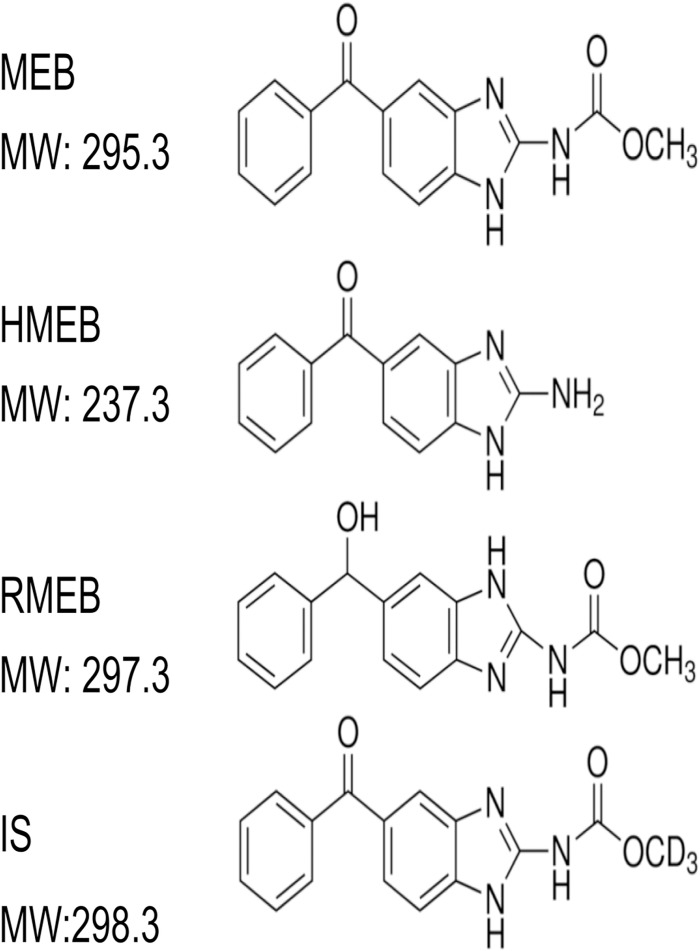
Molecular structures of examined benzimidazoles.

For the experiment, muscle samples of pork, chicken, and horse were collected from the markets in major cities in Korea (Seoul, Busan, Incheon, Daegu, Gwangju, Daejeon, Ulsan, and Jeju). The geographical designation as well as names of the markets are provided in the supplementary information (Table A in S1 File). All the muscle samples were purchased as a packaged meat from the markets, but horse muscle samples were collected from the six restaurants as horse meat could not be purchased from the markets. Analytical standards of MEB, HMEB, RMEB, and the internal standard (IS) 5-hydroxy mebendazole-d3 were purchased from Dr. Ehrenstorfer (Augsburg, Germany). The molecular structures of these substances are shown in Fig 1. Acetonitrile (ACN), hexane, formic acid, ammonium formate, methanol and ethyl acetate at HPLC-grade were purchased from Sigma-Aldrich (St. Louis, MO, USA). Dimethyl sulphoxide (DMSO) and sodium hydroxide (NaOH) were supplied by Daejung chemical and metals Co., Ltd. (Siheung, Gyeonggi, South Korea). Water was purified with a milli-Q plus apparatus (Millipore, Bedford, MA, USA).

### Standard solutions

Stock standard solutions (1000 μgmL^-1^) were prepared by dissolving 10 mg of each compound in 10 mL of DMSO. Working standard solutions (100 μgmL^-1^) were prepared by dissolving 1 mL of stock standard solution in 9 ml of ACN. A 1.0 μgmL^-1^ mixed standard solution was prepared by combining 1.0 mL of each working standard solution and dilute with 100 mL of water containing 10 mM ammonium formate and methanol (50:50, v/v). A 1.0 μgmL^-1^ labeled IS was prepared separately. Stock solutions were stored at 4 ^o^C and working and mixed standard solutions were freshly prepared by appropriate dilution of aliquoted stock solutions.

### Sample preparation

Muscle samples of pork, chicken, and horse were collected from the markets in major cities in Korea (Seoul, Busan, Incheon, Daegu, Gwangju, Daejeon, Ulsan, and Jeju). Samples were ground using a high-speed food blender and stored at -20 ^o^C before the analysis. Each ground sample (2 g) was aliquoted into 50 mL centrifuge tube. If necessary, MEB, HMEB, RMEB, and/or IS solutions were added at this stage. Then the sample was homogenized with 10 mL distilled water and made alkaline with 1 mL of 1M NaOH. Analytes were extracted with ethyl acetate (20 mL) on a shaker for 10 minutes. After separation by centrifugation at 5000 rpm for 5 minutes, the supernatant was transferred into an evaporation tube. The extraction procedure was repeated twice with 10 mL of ethyl acetate. The collected organic fractions were rapidly evaporated to dry at 60 ^o^C under a stream of nitrogen using XcelVap automated evaporation system (Horizon technology, New Hampshire, USA). The residue was dissolved in 1.2 mL mobile phase (water containing 10 mM ammonium formate and methanol at 50:50 v/v). The sample was further washed by washing with 2 mL *n*-hexane. The upper hexane layer was removed and the sample was filtered with a 0.2 μm polytetrafluorethylene (PTFE) before being injected into the LC column.

### LC-MS/MS analysis

The liquid chromatography system (Shiseido Co., Ltd., Tokyo, Japan) was equipped with a solvent delivery pump (SI-2/3101), an auto sampler (SI-2/3133), and a column oven (SI-2/3004). Analytes were separated with a Unison UK C18 column (3 μm, 100 mm x 2 mm id, Imtakt, USA). The temperature of the column oven was set at 40 ^o^C. Gradient elution program was applied with mobile phase consisting of 10 mM ammonium formate (phase A) and methanol (phase B). The flow rate and injection volume were 0.25 mL/ min and 5 μl, respectively. The gradient profile is shown in Table 2.

**Table 2:** Gradient elution profile.

Time(min)	A(%)^a^	B(%)^b^
0	70	30
0.5	70	30
4.0	5	95
7.0	5	95
7.1	70	30
10.0	70	30

^a^mobile phase A: 10 mM Ammonium formate.

^b^mobile phase B: methanol.

MS/MS analysis was performed using API 4000 triple quadrupole mass spectrometer instrument (AB Sciex, MA, USA) operated in positive (ESI^+^) electrospray ionization mode. Analysis was performed with nitrogen using the following settings: curtain gas with a pressure of 20 psi, ion source gas 1 with a pressure of 50 psi, and ion source gas 2 with a pressure of 50 psi. The source temperature and ion spray voltage (IS) were set at 500°C and 5500 V, respectively. The mass spectrometer was operated in multiple reaction monitoring (MRM) mode and one precursor ion and two product ions for each target compound was selected. The mass spectrometer was operated at unit mass resolution for both Q1 and Q3 in MRM mode using a dwell time of 150 msec for all analytes. The monitored protonated cations and optimized MS operating parameters of MEB, HMEB, RMEB, and IS are summarized in Table 3. The MRM-generated sample data of the transition from parent ion to most abundant daughter ion were evaluated using an internal standard procedure based on matrix calibration curves. Quantification was conducted by internal calibration. Results were calculated using AB Sciex Analyst software (version 1.6).

**Table 3:** Summary of diagnostic ions and mass operating parameters.

Compound	Precursor ion(m/z)	Product ions(m/z)	Collision energy (eV)
Mebendazole (MEB)	296	264	31
		105	49
5-OH-Mebendazole (RMEB)	298	160	59
		266	49
Mebendazole-amine (HMEB)	238	77	49
		105	33
5-OH-Mebendazole-d3	301	79	55
		266	55

### Method validation

The developed method was validated according to European Legislation 2002/657/EC [15]. For validation, following performance parameters were determined: specificity, linearity, recovery, the precision (repeatability and within-laboratory reproducibility), decision limit (CCα), detection capability (CCβ), limit of detection (LOD), and limit of quantification (LOQ).

To determine the specificity, 20 blank muscle samples for each matrix from different origins were analyzed. The analytes were identified by matching retention times of peaks to the values of the corresponding standard analyzed under the same experimental conditions. For confirmation purpose, specific fragmentation pattern of individual analyte (Table 3) was used to distinguish the analyte from the matrix interferences, thus allowing for greater evidence in compound identification.

The linearity to determine MEB and its metabolites in the muscle tissues of pork, chicken, and horse was tested at concentrations of 5, 10, 30, 60, and 90 μgkg^-1^. Concentration of the IS along the calibration curve was maintained constant at 30 μgkg ^-1^. Three series of calibration curves were prepared by plotting the peak area against analyte concentrations expressed as μgkg^-1^. The linear regression equations and correlation coefficients were calculated for the curves and used for calculating the concentrations of analytes. The linearity of calibration curves in pork was demonstrated with *F*-test lack of fit conducted in four replicates at concentrations of 5, 10, 30, 60, and 90 μgkg^-1^.

The EU requirements describe that the recovery has to be determined by experiments using six fortified blank matrices at 0.5, 1, and 1.5 times the permitted limit for each [14]. For validation study, this condition stipulated in requirements cannot be applied because the MRL of MEB for pork, chicken, and horse muscle is set at 60 μgkg^-1^ for the sum of concentration of parent compound (MEB) and its metabolites (RMEB, HMEB). The MRL of 60 μgkg^-1^ has to be divided among MEB, RMEB, and HMEB. Therefore, a validation limit of 15 μgkg^-1^ for pork, chicken, and horse muscle was chosen. Two higher concentrations at 30 and 60 μgkg^-1^ were also chosen. Blank samples were fortified with mixed standard solutions of MEB and its metabolites. Total of six replicates at each level were analyzed to calculate the recovery (expressed as percentile).

To evaluate the precision of this analytical method, its repeatability and within-laboratory reproducibility, were determined. To determine its repeatability, six replicates at each spiking level were analyzed in the same way for the recovery. Standard deviation (SD) and coefficient of variation (CV, %) were calculated for each level. To evaluate the within-laboratory reproducibility at each level, the analyses were performed with six replicates on three different days by two different operators using mixed standard solutions prepared daily followed by overall SD and CV calculation.

The decision limit (CCα) and detection capability (CCβ) were calculated using within-laboratory reproducibility validation results according to the procedure described in the 2002/657/EC decision [14]. CCα of the described method for mebendazole and its metabolites are defined as the mean values of the calculated concentrations by determining twenty blank samples spiked at the validation limit of 15 μgkg^-1^ with the analytes plus 1.64 times of the corresponding standard deviations. CCβ was calculated as the respective CCα values plus 1.64 times of the corresponding standard deviations of the measured concentrations by analyzing twenty blank samples spiked at the respective CCα with the analytes.

Limit of detection (LOD) and limit of quantification (LOQ) were calculated based on the signal to noise ratio (S/N = 3 for LOD and S/N = 10 for LOQ) of the chromatograms of 20 blank muscle samples of pork, chicken, and horse. To verify LOD and LOQ values, pork, chicken, and horse samples were spiked with MEB+RMEB+HMEB at the level of 0.5 μgkg^-1^.

## Results and Discussion

### Optimization of sample preparation

**Fig 2:**
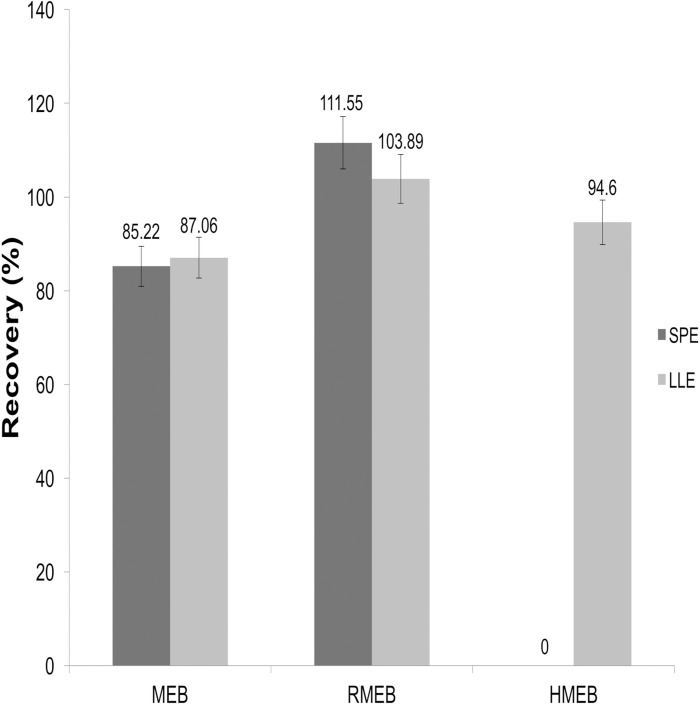
Effect of sample purification method on MEB, HMEB, and RMEB recovery in pork spiked at the concentration of 30 μgkg^-1^.

The procedure of purification in mebendazole analysis method of the current Korean Food Standards Codex is based on SPE by using a polypropylene centrifuge tube (15 ml) containing 900 mg MgSO_4_ and 150 mg PSA. This method showed low recovery rate for the detection of mebendazole residues, especially in the case of HMEB in pork (Fig 2). This could be due to high protein and fat contents in this type of matrix that can often interfere with the analytical procedure [15]. In the paper, anti-oxidizing agents such as BHT and ascorbic acid were used to prevent the oxidation of mebendazole to metabolites [16]. However, they failed to show good results for the recovery rate of mebendazole residues from milk. Therefore, we decided to change the sample preparation method from SPE to LLE. Sample preparation procedure described in this study was based on the LLE method reported by De Ruyck *et al* [17]. Specifically, the sample was first alkalized using NaOH followed by target analytes extraction with ethyl acetate. The extract was then defatted with hexane. The recovery rates after ethyl acetate extraction for MEB, RMEB, and HMEB were improved from 85.22 to 87.06, 111.55 to 103.89, and 0 to 94.6, respectively, in pork spiked at concentration of 30 μgkg^-1^ (Fig 2). Zhu *et al* have showed similar result by showing that extracted animal samples are much cleaner after ethyl acetate extraction, although SPE cartridges proved to be preferable for purifying crude extracts [18].

### Optimization of the LC-MS/MS

**Fig 3:**
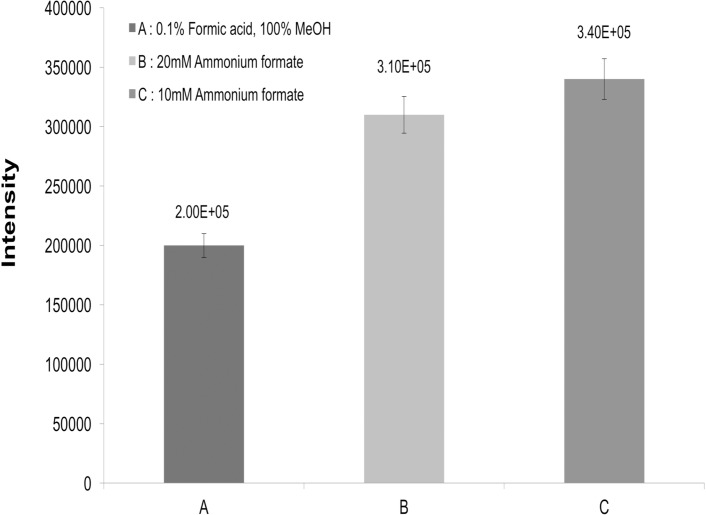
Changes in the intensity of MEB, HMEB, and RMEB in pork spiked at the concentration of 30 μgkg^-1^ with different additives (0.1% Formic acid, 20 mM Ammonium formate, and 10 mM Ammonium formate).

The mobile phase composition and additives may significantly influence the response of a solute because the ionization by an ESI source occurs in the solution state. In this context, Zue *et al* [18] have reported that the responses of methanol-water + 0.1% formic acid are greater than those obtained when acetonitrile-water + 0.1% formic acid is used to provide protons in positive mode of LC-MS/MS aided protonation of benzimidazoles. Many authors have used ammonium formate and formic acid in mobile phase to increase ionization and achieve maximum sensitivity [10, 11, 15, 17–19]. In this respect, three reagents (0.1% formic acid, 10 mM ammonium formate, and 20 mM ammonium formate) with methanol were tested in this study (Fig 3). The mobile phase consisting of water containing 10 mM ammonium formate and methanol resulted in wider separation than that with water containing 0.1 formic acid and methanol. Whelan *et al* [19] have reported similar results. The gradient given in Table 2 was optimized to provide the maximum separation possible in a minimum time period.

**Fig 4:**
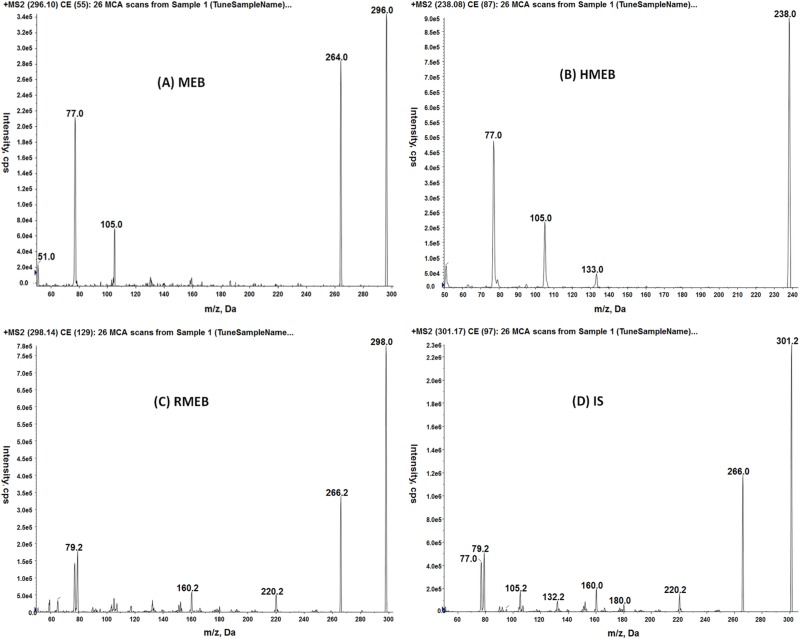
MS/MS (MS2 scan) spectra of (A) MEB, (B) RMEB, (C) HMEB and (D) IS.

Mass spectrometry conditions were initially optimized by infusing mixed standards of MEB + RMEB + HMEB + IS at concentration of 100 μgL^-1^ and tuning collision energies by using mobile phase consisting of water containing 10 mM ammonium formate and methanol (50:50, v/v). Positive electrospray ionization mode was found to be suitable for all the analytes and IS. The product ion spectra (MS/MS) of MEB, RMEB, HMEB and IS are shown in Fig 4. Diagnostic ions and MS operating parameters are summarized in Table 3. Determination of mebendazole, its metabolites, and IS were performed by MRM. The detection of analytes in standard solutions and spiked muscle tissues of pork, chicken, and horse was performed by two MRM transitions (two transitions from parent ion (*M*+*H*)+ to the two most abundant daughter ions).

### Validation of the analytical method

**Fig 5:**
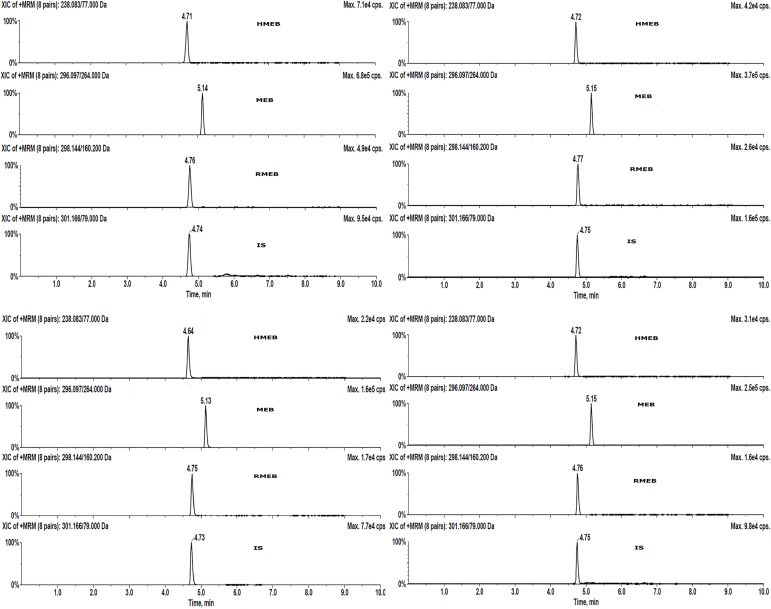
HPLC-MS/MS chromatograms of (A) standard, (B) pork, (C) chicken, and (D) horse fortified with MEB, RMEB, HMEB, and IS at 30 μgkg^-1^.

Results of validation for each analyte, the sum of parent compound, and its metabolites are presented in Table 4. The specificity of the method was demonstrated by checking interfering peaks at the retention time of target analytes. The results showed that there were no interfering peaks co-eluted with target analytes. The developed LC-MS/MS method is capable of separating all analytes under the given gradient condition within 10 min, demonstrating that the method could be applied to monitor the residues in different livestock samples. Representative chromatograms of each fortified samples are shown in Fig 5.

**Table 4:** Validation results of the multi-residue method for the determination of mebendazole in pork, chicken, and horse muscles.

	Analyte	Internal standard	Spiking level, μg/kg			Recovery, %			Repeatability, CV, %			Within-laboratory reproducibility, CV, %			CCα	CCβ
			Ⅰ	Ⅱ	Ⅲ											
Pork	MEB	HMEB-D3	15	30	60	101.28	94.08	91.06	4.46	3.43	11.23	5.24	5.75	10.38	16.43	17.96
	RMEB					99.33	93.81	91.06	5.59	4.82	10.52	6.85	6.93	10.72		
	HMEB					97.36	91.44	88.35	4.32	4.55	11.02	6.67	7.41	10.44		
	∑MEB					99.32	93.11	90.16	4.79	4.27	10.92	6.64	5.73	6.16		
Chicken	MEB	HMEB-D3	15	30	60	93.86	89.85	91.03	6.41	6.35	5.22	8.80	5.49	6.95	15.84	17.94
	RMEB					91.00	91.63	93.91	6.72	5.43	5.73	8.81	6.18	7.51		
	HMEB					89.81	90.86	94.53	8.90	3.50	6.46	9.89	4.56	8.87		
	∑MEB					91.56	90.78	93.16	7.35	5.09	5.80	6.63	4.27	5.78		
Horse	MEB	HMEB-D3	15	30	60	96.42	100.25	94.56	7.65	9.94	8.18	6.80	8.39	9.68	15.97	17.60
	RMEB					90.56	88.90	86.30	4.95	5.94	9.43	8.27	10.45	10.42		
	HMEB					100.03	96.63	95.23	5.86	8.52	10.06	5.71	8.65	10.17		
	∑MEB					95.67	95.26	92.03	6.15	8.13	9.23	5.68	7.89	5.72		

To compensate for compound loss during sample preparation, measurements of linearity were carried out with matrix-matched standard solutions at concentration of 5–90 μgkg^-1^. The r^2^ values for all analytes were greater than 0.999 as shown in Table 5 and good linearity was observed (Figure A in S1 File). Pursuant to Analytical Methods Committee (AMC), a value of correlation coefficients close to unity is not necessarily the outcome of a linear relationship. In this context, the test for the lack of fit should be verified [20]. Therefore, linearity of calibration curves was demonstrated with *F*-test lack of fit and the working range was established. ANOVA results for the linearity of MEB, RMEB, and HMEB in pork are shown in Table 6. The *F* value for the lack of fit was smaller than the tabulated *F* value at the 95% confidence level (α = 0.05). According to the ANOVA test, the linear regression showed no lack of fit.

**Table 5:** Linear regression equations and correlation coefficients of standard curves for mebendazole and its metabolites.

	Analyte^a^	Linear equation^b^	Correlation coefficient value (r^2^)
Pork	MEB	Y = 0.167X-0.128	0.999
	RMEB	Y = 0.0232X-0.0207	0.999
	HMEB	Y = 0.0151X-0.0103	0.999
Chicken	MEB	Y = 0.156X-0.131	0.999
	RMEB	Y = 0.0209X-0.0226	0.999
	HMEB	Y = 0.0149X-0.00269	0.999
Horse	MEB	Y = 0.156X-0.131	0.999
	RMEB	Y = 0.0209X-0.0226	0.999
	HMEB	Y = 0.0149X-0.00269	0.999

^a^The concentration ranged from 5 to 90 ugkg^-1^.

^b^Y = AX + B, where Y is peak area and X is the concentration of the analyte.

**Table 6:** ANOVA results for the linearity of MEB, RMEB, and HMEB.

Analyte	Source of variation	Sum of Squares	Degree of Freedom	Mean Sum of Squares	F-ratio
MEB	Regression	2.87 x 10^13^	1	2.87 x 10^13^	0.0025
	Residual	5.14 x 10^13^	18	2.86 x 10^12^	
	Lack of fit	2.56 x 10^10^	3	8.53 x 10^09^	
	Pure error	5.14 x 10^13^	15	3.43 x 10^12^	
	Total	8.01 x 10^13^	19		
RMEB	Regression	8.47 x 10^12^	1	8.47 x 10^12^	0.1795
	Residual	2.11 x 10^11^	18	1.17 x 10^10^	
	Lack of fit	7.33 x 10^09^	3	2.44 x 10^09^	
	Pure error	2.04 x 10^11^	15	1.36 x 10^10^	
	Total	8.68 x 10^12^	19		
HMEB	Regression	3.36 x 10^12^	1	3.36 x 10^12^	0.0186
	Residual	6.72 x 10^11^	18	3.73 x 10^10^	
	Lack of fit	2.48 x 10^09^	3	8.28 x 10^08^	
	Pure error	6.69 x 10^11^	15	4.46 x 10^10^	
	Total	4.03 x 10^12^	19		

The critical value of F-ratio is 3.2873 at alpha = 0.05.

The recovery rates of the procedure were determined by spiking blank samples with three different levels (15, 30, and 60 μgkg^1^) of standard materials in six replicates. For all analytes, recovery rates were high (in the range of 86–101%) and the precision level was relatively good (Table 4). The repeatability and within-laboratory reproducibility were found to be in the range of 3.4–11.2% (CV, %) and 4.2–10.7% (CV, %), respectively. However, a standard analytical LC-MS/MS method for mebendazole residues using DMSO extraction followed by SPE purification depicted in Korean Food Standards Codex showed very low recovery rates (0%) for HMEB but relatively high (85–111%) for MEB and RMEB in pork (Fig 2) [6]. These results indicated that LC-MS/MS with LLE can be a precise and accurate alternative for simultaneous determination of MEB and its metabolites in pork, chicken, and horse. The calculated CCα and CCβ values (CCα / CCβ) were 16.4 / 17.9 (pork), 15.8 / 17.9 (chicken), and 15.9 / 17.6 (horse) μgkg^-1^. The decision limit (CCα) and detection capability (CCβ) were calculated using within-laboratory reproducibility validation results according to the procedure described in the 2002/657/EC decision [14].

The more commonly used LOD and LOQ were also studied. Limit of detection was calculated with higher than 3 of the S/N ratio and LOQ was calculated with higher than 10 of the S/N ratio. The result showed that the calculated LOD and LOQ were about 0.07 μg/kg and 0.2 μg/kg, respectively.

### Application of the developed method

The developed method was applied for the analysis of 86 commercially available animal based foods such as pork, chicken, and horse collected from the markets located in major cities (Table 7). The collected food samples consisted of 45 pork (52%), 35 chicken (41%), and 6 horse (7%) meat. All samples were processed according to the method described above and the analytical results showed that none of the sample contained any detectable amount of mebendazole residues except one sample in chicken. However, the detection level of mebendazole residues in chicken was below the MRL (maximum residue limits).

**Table 7:** Summary of purchase region, numbers of collected samples, and mebendazole residues results.

Cities	Pork	Chicken	Horse	Total
Seoul	19 (ND^a^)	14 (1D^b^)	–	33 (1D)
Busan	7 (ND)	6 (ND)	–	13 (ND)
Incheon	5 (ND)	5 (ND)	–	10 (ND)
Daegu	5 (ND)	3 (ND)	–	8 (ND)
Gwangju	3 (ND)	3 (ND)	–	6 (ND)
Daejeon	3 (ND)	2 (ND)	–	5 (ND)
Ulsan	3 (ND)	2 (ND)	–	5 (ND)
Jaeju	–	–	6 (ND)	6 (ND)
Total	45 (ND)	35 (1D)	6 (ND)	86 (1D)

^a^ND: not detected.

^b^1D: mebendazole residue detected from 1 sample.

## Conclusion

A simple and effective LC-MS/MS method was developed for simultaneous determination of mebendazole, its hydrolysed metabolite, and reduced metabolite in pork, chicken, and horse muscles. Mebendazole residues were successfully separated by LC-MS/MS with LLE. Compared with conventional methods, the developed method resulted in satisfactory validation characteristics with respect to specificity, accuracy, precision, analytical limits, and applicability. Therefore, this proposed screening method has great potential for the control of mebendazole residues in livestock products.

## Supporting Information

S1 File Summary of the geographical designation and the names of the markets from where the pork, chicken, and horse muscle samples were collected. All the muscle samples were domestic origin except 8 pork samples (3 from USA, 1 from Poland, 2 from Canada, and 1 from France) (Table A). Calibration curve of MEB, RMEB and HMEB in (A) pork, (B) chicken an (C) horse muscle (Figure A).
(DOCX)
