# Supplementary material for: A Liquid Chromatography – Tandem Mass Spectrometry Approach for the Identification of Mebendazole Residue in Pork, Chicken, and Horse
Source: PLoS One. 2017 Jan 13;12(1):e0169597. doi: 10.1371/journal.pone.0169597 (PMC5234820; doi:10.1371/journal.pone.0169597)
Supplement: S1 File — All the muscle samples were domestic origin except 8 pork samples (3 from USA, 1 from Poland, 2 from Canada, and 1 from France) (Table A). Calibration curve of MEB, RMEB and HMEB in (A) pork, (B) chicken an (C) horse muscle (Figure A). (DOCX) [file pone.0169597.s001.docx]

**Supporting information**

**A liquid chromatography – tandem mass spectrometry approach for the identification of mebendazole residue in pork, chicken, and horse**

Ji Sun Lee ^1 2^, Soo Hee Cho ^3^, Chae Mi Lim ^4^, Moon Ik Chang ^5^, Hyun Jin Joo ^4^, Hojae Bae ^6*^, Hyun Jin Park ^2 7*^

^1^Imported Food Analysis Division, Seoul Regional Food and Drug Administration, 212 Mokdongjungang-ro, Yangcheon-Gu, Seoul, 07978, Republic of Korea

^2^Department of Biotechnology, College of Life Sciences and Biotechnology, Korea University, 5-Ka, Anam-Dong, Sungbuk-Gu, Seoul, 136-701, Republic of Korea

^3^Korea Health Supplements Association Sub. Korea Health Supplements Institute, B-dong 101, 700 Daewangpangyo-ro, Bundang-Gu, Seongnam-si, Gyeonggi-do, 13488, Republic of Korea

^4^Ministry of Food and Drug Safety, 187 Osongsaengmyeong2(i)-ro, Osong-eup, Heungdeok-gu, Chungju-si, Chungcheonbuk-do, 28159, Republic of Korea

^5^Pesticide and Veterinary Drug Residues Division, Food Safety Evaluation Department, National Institute of Food and Drug Safety Evaluation, Ministry of Food and Drug Safety, 187 Osongsaengmyeong2(i)-ro, Osong-eup, Heungdeok-gu, Chungju-si, Chungcheonbuk-do, 28159, Republic of Korea

^6^College of Animal Bioscience and Technology, Department of Bioindustrial Technologies, Konkuk University, Hwayang-dong, Kwangjin-gu, Seoul 143-701, Korea

^7^Department of Packaging Science, Clemson University, Clemson, SC 29634-0320, USA

* Corresponding author

Email: hojaebae@konkuk.ac.kr; hjpark@korea.ac.kr

**S1 File**. Summary of the geographical designation and the names of the markets from where the pork, chicken, and horse muscle samples were collected. All the muscle samples were domestic origin except 8 pork samples (3 from USA, 1 from Poland, 2 from Canada, and 1 from France) (Table 7). Calibration curve of MEB, RMEB and HMEB in (A) pork, (B) chicken an (C) horse muscle (Table 5).

**Table A.** Summary of the geographical designation and the names of the markets from where the pork, chicken, and horse muscle samples were collected.

| **City** | **Market** | **Geographical Coordinates** | **Collection Date** |
| --- | --- | --- | --- |
| Seoul | Lotte mart (Gimpo airport store) | 37˚39’24.31”N 126˚40’10.96”E | 2013.05.31 |
|  | Lotte mart (Seoul station store) | 37˚33’20.82”N 126˚59’13.98”E |  |
|  | Lotte department store (Jamsil store) | 37˚30’41.76”N 127˚5’46.75”E |  |
|  | Homeplus (Dongdaemun store) | 37˚34’28.24”N 127˚2’19.53”E |  |
|  | Hanaro club (Yangjae store) | 37˚27’46.78”N 127˚2’35.18”E |  |
| Busan | Shinsegae department (Centum city store) | 35˚10’7.33”N 129˚7’47.09”E | 2013.09.03 |
|  | Mega mart (Gijang store) | 35˚15’16.49”N 129˚13’19.15”E |  |
| Incheon | E-mart (Incheon store) | 37˚26’34.45”N  126˚42’03.48”E | 2013.07.04 |
|  | E-mart traders (Ganseok store) | 37˚28’59.04”N 126˚39’38.01”E (Songlim store) | 2013.07.18 |
|  | Homeplus (Ganseok store) | 37˚28’10.25”N 126˚41’21.10”E | 2013.08.13 |
| Daegu | Lotte department store (Daegu store), | 35˚52’32.55”N 128˚35’42.96”E | 2013.07.26 |
|  | Homeplus (Daegu store) | 35˚52’52.96”N 128˚35’44.80”E |  |
| Gwangju | Lotte supermarket (Jinwall store) | 35˚07’28.08”N 126˚53’51.55”E | 2013.07.18 |
|  | E-mart (Sangmu store) | 35˚09’20.47”N 126˚51’15.06”E |  |
|  | Homeplus (Iksan store) | 35˚57’31.97”N 126˚58’20.46”E | 2013.07.19 |
|  | Homeplus (Jinwall store) | 35˚09’41.07”N 126˚55’00.14”E (Gwangju gyelim store) | 2013.07.18 |
| Daejeon | Homeplus (Munwha store), | 36˚19’13.04”N 127˚24’27,37”E | 2013.06.27 |
|  | Costco (Seodaejeon store) | 36˚19’26.21”N 127˚24’12.44”E |  |
| Ulsan | Hyundai department store (Ulsan store) | 35˚32’22.66”N 129˚20’08.79”E | 2013.09.13 |
|  | Lotte mart (Ulsan store) | 35˚32’15.36”N 129˚19’50.46”E |  |
| Jeju | Halasan green pork restaurant, | 33˚30’06.35”N 126˚30’47.42”E | 2013.07.04 |
|  | Donma agricultural corporation | 33˚28’49.58”N 126˚29’49.03”E |  |
|  | Gowooni restaurant | 33˚28’54.75”N 126˚28’13.84”E |  |
|  | Mokjangwon Basme | 33˚24’21.60”N 126˚46’36.14”E |  |
|  | chungjunhae restaurant | 33˚29’12.55”N 126˚31’48.22”E |  |

**Figure A**. Calibration curve of MEB, RMEB and HMEB in (A) pork, (B) chicken an (C) horse muscle (Table 5).

**Pork**

(A)


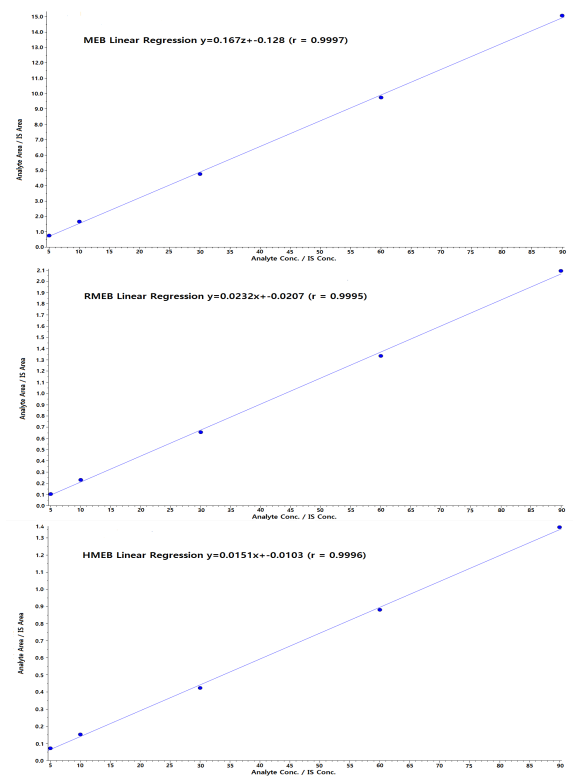


(B)

**Chicken**

**
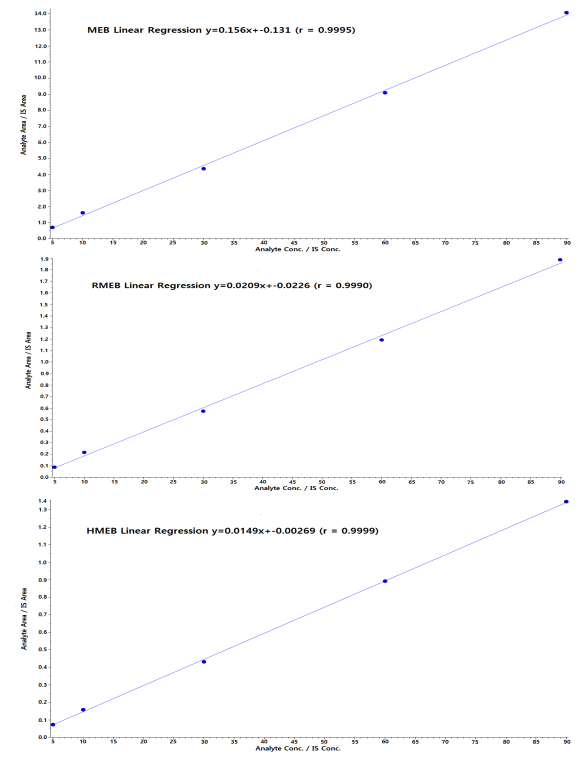
**

**(C)**

**Horse**

**
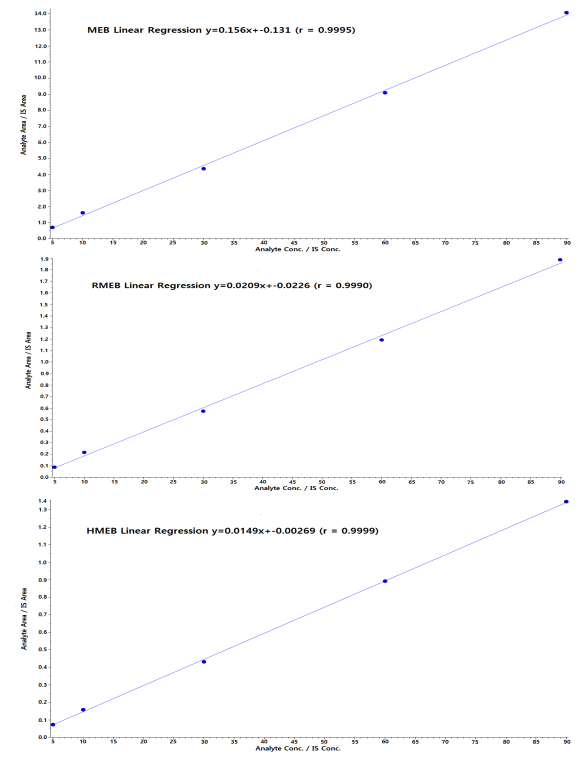
**
